# Serum TLR2 and TLR9 in Prostate Cancer Patients in Relation to EBV Status

**DOI:** 10.3390/ijms25169053

**Published:** 2024-08-21

**Authors:** Dominika Sikora, Jacek Kiś, Ewa Stępień, Bartłomiej Drop, Małgorzata Polz-Dacewicz

**Affiliations:** 1 Department of Virology with Viral Diagnostics Laboratory, Medical University of Lublin, 20-093 Lublin, Poland; ewa.stepien@umlub.pl (E.S.); malgorzata.polz-dacewicz@umlub.pl (M.P.-D.); 2 1st Clinical Military Hospital with Outpatient Clinic in Lublin, 20-049 Lublin, Poland; jacekkis@gmail.com; 3 Department of Medical Informatics and Statistics with e-Health Lab, Medical University of Lublin, 20-090 Lublin, Poland; bartlomiej.drop@umlub.pl

**Keywords:** prostate cancer, Epstein–Barr virus, TLR-2, TLR-9

## Abstract

The relationship between Toll-like receptors (TLRs) and prostate cancer (PCa) is complex due to the presence of the Epstein–Barr virus (EBV) infection, which has been identified as a predisposing factor for some cancers, including PCa. The present study aims to investigate these complex links by examining the levels of selected TLRs and the potential impact of EBV infection on PCa. Therefore, we examined the serum of patients with PCa. The study compared EBV(+) patients to risk groups, the Gleason score (GS), and the T-trait. Additionally, the correlation between TLR and antibody levels was examined. The results indicated that higher levels of TLR-2 and TLR-9 were observed in more advanced PCa. The findings of this study may contribute to a deeper understanding of the role of viral infections in PCa and provide information on future strategies for the diagnosis, prevention, and treatment of these malignancies.

## 1. Introduction

Prostate cancer (PCa) is an important global health issue. A total of 1,465,854 men were diagnosed with it in 2022. This represents a substantial increase from the previous year [1,2]. According to the World Health Organization (WHO), it is the fifth leading cause of cancer-related mortality worldwide. In Poland, the incidence of this type of cancer is increasing, and the Ministry of Health and the National Health Fund forecasts that this trend will continue. In Poland, approximately 15,000 men are diagnosed with PCa each year, and it is expected that this number will soon reach approximately 20,000 men per year [3].

PCa usually begins in the peripheral part of the prostate. PCa is often initially asymptomatic, and the symptoms resemble benign organ hyperplasia. Prostate issues may result in a sudden urge to urinate, frequent urination, a burning sensation upon urination, or a sensation that the urine has not been fully expelled. Its aggressive nature and asymptomatic early stage make it a severe problem requiring attention and intervention [4]. When invasive cancer infiltrates the surrounding tissues and organs, it spreads through the blood and lymphatic vessels, often metastasizing to the bones [5].

The diagnosis is usually based on a laboratory test during which the prostate-specific antigen (PSA) level is measured in blood serum [5,6]. This biomarker is widely used in PCa screening because it enables a quick and straightforward diagnosis of the disease. An increased PSA level may also indicate prostate enlargement [5,6], which is why the diagnosis of PCa is often based on a microscopic examination. This test is based on the Gleason classification, which assesses tumour tissue structure [5]. It is important to note that the search for new biomarkers is ongoing and may lead to further advances in diagnosing and treating PCa. It is worth emphasizing that the prognosis for PCa depends on both the stage of the disease and the treatment used.

PCa morbidity and mortality rates vary widely around the world. Many factors influence the development of PCa, including immutable characteristics such as age, race, familial and germline mutations, and variable factors such as metabolic syndrome, obesity, and smoking [7,8]. Being able to recognize and understand these factors and their impact on the risk of PCa progression is crucial, as it may help us gain insights into the development of PCa and allow for more effective treatment strategies to emerge. This therefore requires further research. It is necessary to increase awareness of the impact of risk factors on PCa and encourage healthy lifestyles to reduce risk. Many authors emphasize the potential role of persistent viral infections in developing PCa [9,10].

The Epstein–Barr virus (EBV) belongs to the *Herpesviridae* family, which can infect most of the human population worldwide. It is the first known human virus with carcinogenic potential. It has been associated with the development and progression of various B-cell malignancies, such as Burkitt’s lymphoma and Hodgkin’s lymphoma, as well as epithelial malignancies, such as gastric cancer and oropharyngeal cancer (NPC) [11,12]. The association of EBV with cancers is well-established and widely known in the medical literature. PCa has been scientifically proven to contain EBV DNA [6]. After the initial infection, the virus enters the latent phase in infected cells. It may be periodically reactivated, leading to the lytic cycle with viral transmission, which may influence the pathogenesis of EBV-related cancers. Although the transition mechanism from the latent phase to the lytic phase is still not fully understood, it is an active area of research [13].

**Figure 1:**
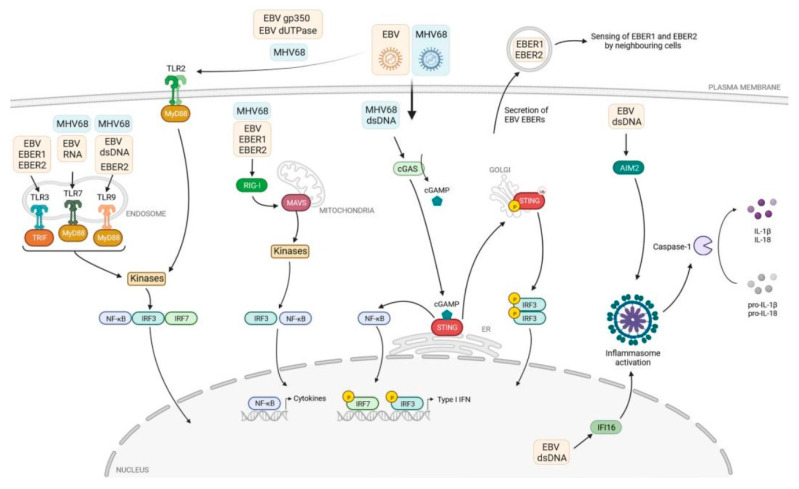
The immune response and TLR signalling pathway [18].

Toll-like receptors (TLRs) are a class of pathogen recognition receptors (PRRs) that play a pivotal role in the innate immune system. They are responsible for identifying pathogen-associated molecular patterns (PAMPs) and damage-associated molecular patterns (DAMPs) [14,15]. TLRs can recognize many pathogens, including viruses and cancer cells. These receptors are necessary for the proper functioning of the immune system. TLRs are expressed on various immune and non-immune cells, including dendritic cells, macrophages, T-cell subsets, B-cells, epithelial cells, and fibroblasts. TLRs can be classified into two distinct groups based on their subcellular localization. Cell membrane TLRs include TLR-1, -2, -4, -5, -6, and -10, expressed in their active form on the cellular surface and known as ‘membrane’ or ‘surface’ TLRs, while TLR-3, -7, -8, and -9 intracellular TLRs, expressed within the host cells on the organelle biomembranes, are located in the endosome membrane and are known as ‘endosome’ TLRs [16]. Each type of TLR recognizes its specific ligand(s) and activates the associated signalling pathway either in a MyD88- or TRIF-dependent manner. This activation leads to the secretion of various cytokines that help the host body combat multiple invaders. Furthermore, TLRs play a crucial role in the maturation of dendritic cells (DCs), which link innate and adaptive immune responses [16,17,18]. The role of PRR receptors in recognizing molecular structures of various pathogens, including EBV, was very well illustrated by Rex et al. (Figure 1) [18]. These authors presented the mechanism of virus detection by TLRs, cytosolic RIG-I-like receptors (RLRs), as well as nuclear and cytosolic DNA sensors.

We selected two TLRs from different categories for our study. TLR-2 is involved in the pathogenesis of many diseases, including infectious diseases, inflammation, cancer, and autoimmune diseases [19]. Although there is some evidence that TLR-9 may be involved in the occurrence and development of cancer, the precise role of this receptor in disease remains unclear. Some studies have indicated that TLR-9 is associated with increased tumour malignancy, whereas others have suggested that it contributes to the immune response against the tumour [20,21]. Moreover, Zhao et al. [22] reported that the activation of TLR-2, -4, and -9 in PCa cells facilitates tumour growth, while TLR-3, -4, -5, and -7 may act as tumour suppressors.

The relationship between TLRs and PCa is complex due to the presence of EBV infection, which has been identified as a predisposing factor to certain cancers, including PCa. The convergence of TLR signalling and the presence of EBV suggests interesting links between viral infection and immune system activation. Our study aims to dissect these complex associations by examining the levels of selected TLRs and the potential impact of EBV infection on PCa. We therefore tested the serum from PCa patients (divided into two groups due to the presence of EBV) against that of the Control Group. The EBV(+) patients were compared to the risk groups, to the Gleason scale (GS), and to the T-trait. We also looked at the correlation between the TLRs and the levels of antibodies, which we had already studied in our previous work [23].

A comprehensive understanding of the function of TLRs within the PCa microenvironment paves the way for novel therapeutic interventions and prognostic assessments, thereby facilitating a more precise approach to treating this type of cancer.

## 2. Results

### 2.1. Analysis of Selected Parameters of Patients with PCa and the Control Group

Patients with PCa had statistically significant differences regarding pathological features, including risk groups (according to the EAU classification: low, intermediate, and high), the GS, and the TNM (Table 1).

### 2.2. Evaluation of the Level of TLR-2 and TLR-9 in PCa Patients Compared to the Control Group

In the initial phase of the analysis, we used the findings on the prevalence of EBV as presented in our previous study by Kiś et al. [23]. The study included patients with PCa, with 57 patients classified as EBV(+) and 58 as EBV(−). Furthermore, 40 individuals were included in the Control Group.

Comparison of the EBV(+) and EBV(−) PCa patients with the Control Group revealed significant differences in TLR levels. The mean TLR-2 level in the EBV(+) group was 51.36 ng/mL; in the EBV(−) group, it was 41.07 ng/mL; and in the Control Group, it was 5.48 ng/mL. In contrast, the mean TLR-9 level in the EBV(+) group was 13.60 ng/mL; in the EBV(−) group, it was 11.35 ng/mL; and in the Control Group, it was 3.81 ng/mL. Detailed results of the TLR-2 and TLR-9 analysis are provided in Table S1, within the Supplementary Material.

**Figure 2:**
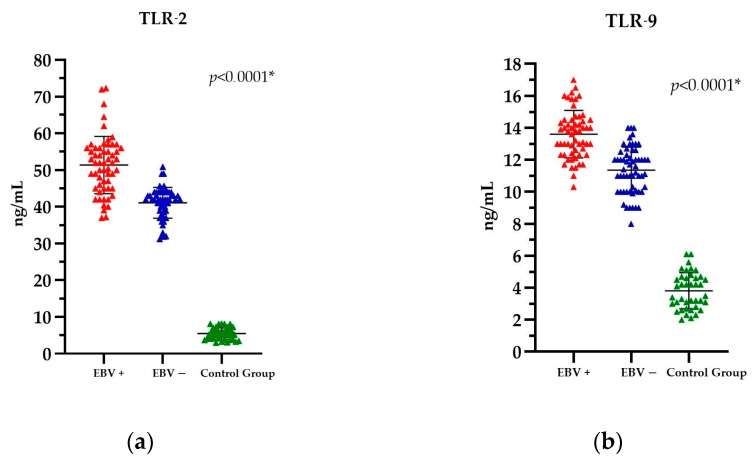
The level of (**a**) TLR-2 and (**b**) TLR-9 in PCa patients EBV(+), EBV(−), and the Control Group. The Kruskal-Wallis test was used to analyse the data; * statistically significant (10^−1^). The results are presented in colour, with red representing PCa EBV(+) patients, blue representing PCa EBV(−) patients, and green representing the Control Group.

Accordingly, the statistical analysis of TLR-2 and TLR-9 levels demonstrated a statistically significant difference between these parameters, *p* < 0.0001 (Figure 2a,b).

Nevertheless, when the data are considered in its entirety, that is, across all three groups (i.e., patients with cancer and EBV(+), patients with cancer and EBV(−), and the Control Group), it becomes evident that patients with PCa and a positive EBV status exhibit the highest levels of both TLR-2 and TLR-9. 

To conduct further analysis, only patients with EBV(+) PCa were included. The relationship between the TLR-2 and TLR-9 levels and the risk group, the GS, and T stage was also investigated.

### 2.3. Evaluation of the Level of TLR-2 and TLR-9 in EBV-Positive PCa Patients in Relation to Risk Group

**Figure 3:**
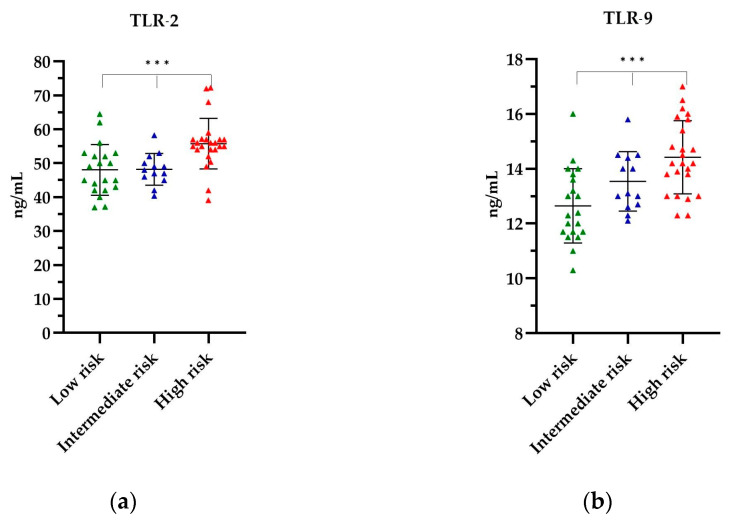
The level of (**a**) TLR-2 and (**b**) TLR-9 in relation to the risk group. The Kruskal–Wallis test was used to analyse the data: (**a**) *p* = 0.0003 and (**b**) *p* = 0.0005; *** statistically significant (10^−3^). The results are presented in color, with green representing low-risk group, blue representing intermediate risk group and red representing the high-risk group.

The levels of TLR-2 and TLR-9 in the PCa patients, stratified according to their respective risk groups, are depicted in Figure 3. The highest mean levels of all TLRs tested were observed in the high-risk group, respectively, as follows: TLR-2 levels were found to be 55.79 ng/mL (*p* = 0.0003) (Figure 3a), while TLR-9 levels were 14.42 ng/mL (*p* = 0.0005) (Figure 3b). The lowest TLR levels were observed in the low-risk group, with 48.09 ng/mL for TLR-2 and 12.65 ng/mL for TLR-9 (Figure 3). The observed differences in the levels of the TLRs tested by the risk group were statistically significant. Further details of the TLR titres are provided in Table S2 of the Supplementary Material.

### 2.4. Evaluation of the Level of TLR-2 and TLR-9 in EBV(+) PCa Patients in Relation to the GS

**Figure 4:**
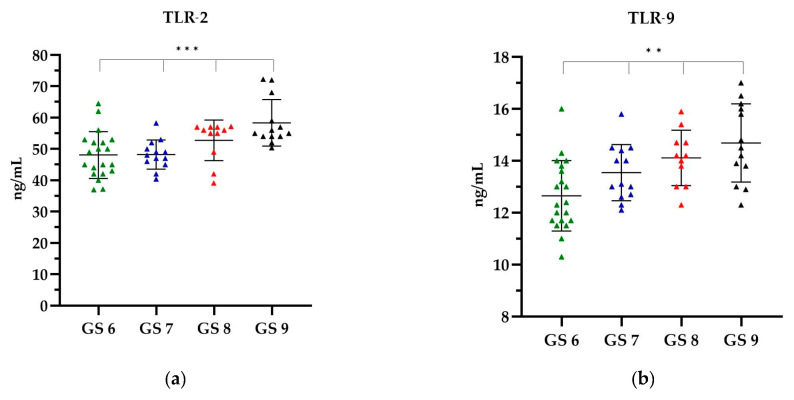
The level of (**a**) TLR-2 and (**b**) TLR-9 in relation to the GS. The Kruskal–Wallis test was used to analyse the data: (**a**) *p* = 0.0006 and (**b**) *p* = 0.0013; ** statistically significant (10^−2^); *** statistically significant (10^−3^). The results are presented in colour, with green representing the GS 6, blue representing the GS 7, red representing the GS 8 and black representing the GS 9.

In the EBV(+) group, categorized according to the GS, when analysing TLR-2, the highest level was observed in GS 9, which was on average 58.36 ng/mL, while the lowest TLR-2 level was observed in GS 6, i.e., 48.09 ng/mL (Figure 4a). With increasing GS, an increase in the level of TLR-9 was observed. The lowest level of TLR-9 was shown in GS 6, i.e., 12.65 ng/mL, while the highest level of 14.68 ng/mL was shown in GS 9 (Figure 4b).

A comparative analysis of the levels of TLR-2 and TLR-9 showed that with the increasing GS, the level of TLRs increased. The observed differences in the concentrations of all TLR types according to the GS were statistically significant (*p* < 0.0001). Further details can be found in the Supplementary Material, Table S3.

### 2.5. Evaluation of the Level of TLR-2 and TLR-9 in EBV(+) PCa Patients in Relation to the T Feature

**Figure 5:**
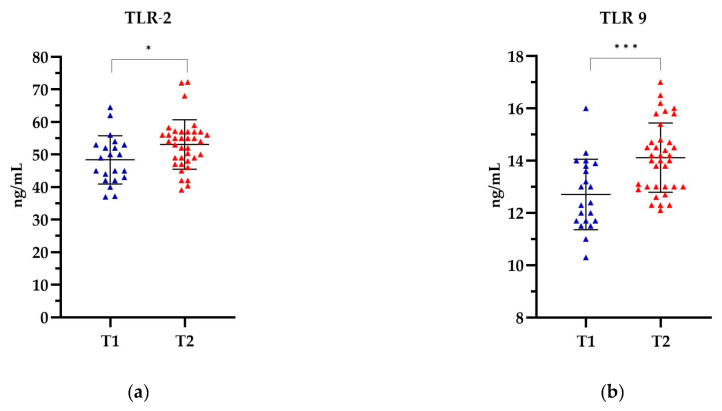
The level of (**a**) TLR-2 and (**b**) TLR-9 in relation to the T stage. The Mann Whitney test was used to analyse the data: (**a**) *p*= 0.0175 and (**b**) *p* = 0.0003; * statistically significant (10^−1^); *** statistically significant (10^−3^). The results are presented in colour, with blue representing T1 and red representing T2.

In patients categorized by the T stage, the highest levels of TLR-2 and TLR-9 were observed in patients in the T2 stage, 53.10 ng/mL and 14.12 ng/mL, respectively, while the lowest levels were observed in the T1 stage, 48.37 ng/mL and 12.71 ng/mL, respectively (Figure 5). The levels of both TLRs were significantly higher in stage T2 compared to the levels of both TLRs in stage T1. The observed differences in TLR levels were statistically significant for TLR-2 (*p* = 0.0175) and for TLR-9 (*p* = 0.0003). Detailed data can be found in Supplementary Table S4.

### 2.6. Correlation between Both Analysed TLRs and Selected Anti-EBV Antibodies

The subsequent stage of the study entailed comparing the TLR results obtained in this study with those of the previous study, which focused on the EBV IgA and IgG antibody results [23]. This study aimed to determine the correlation between the results of the TLR-2 and TLR-9 assays and those of the EBV antibodies of both IgA and IgG classes, namely the Epstein–Barr virus capsid antigen (EBVCA) and Epstein–Barr virus nuclear antigen 1 (EBNA 1). 

**Figure 6:**
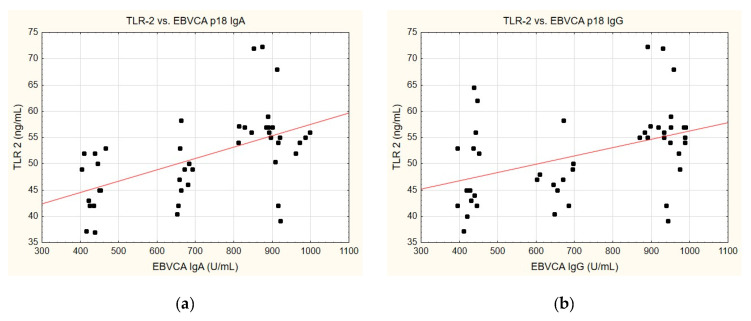

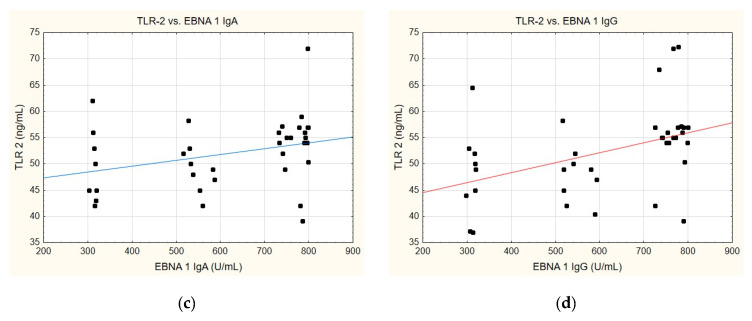
Correlation between TLR-2 and (**a**) EBVCA IgA, (**b**) EBVCA IgG, (**c**) EBNA 1 IgA, (**d**) EBNA 1 IgG the serum levels. Spearman’s rank correlation test (EBVCA IgA and TLR-2 *p* = 0.0003; EBVCA IgG and TLR-2 *p* = 0.0019; EBNA 1 IgA and TLR-2 *p* = 0.0589; EBNA 1 IgG and TLR-2 *p* = 0.0036).

A correlation was identified between the TLR-2 levels and anti-EBVCA (Figure 6a,b) and anti-EBNA (Figure 6c,d). In each case analysed, the increase in TLR-2 levels accompanied the increase in antibody titres. However, only the anti-EBVCA IgA, anti-EBVCA IgG, and anti-EBNA IgG antibodies demonstrated statistical significance. Detailed data can be found in Supplementary Table S5.

**Figure 7:**
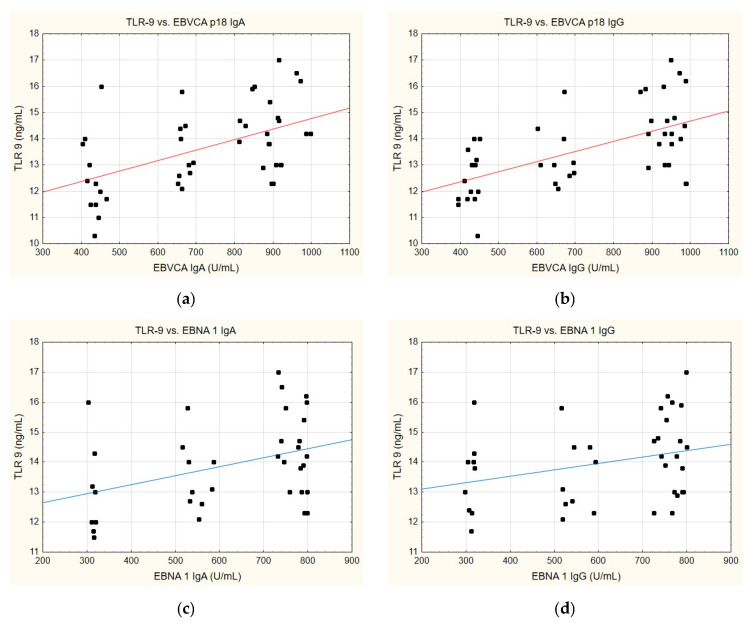
Correlation between TLR-9 and (**a**) EBVCA IgA, (**b**) EBVCA IgG, (**c**) EBNA 1 IgA, (**d**) EBNA 1 IgG the serum levels. Spearman’s rank correlation test (EBVCA IgA and TLR-9 *p* = 0.0007; EBVCA IgG and TLR-9 *p* = 0.0002; EBNA 1 IgA and TLR-9 *p* = 0.1463; EBNA 1 IgG and TLR-9 *p* = 0.0697).

A similar trend can also be discerned when analysing the correlation between TLR-9 levels and the anti-EBVCA and anti-EBNA antibodies (Figure 7). In this instance, a parallel can emerge whereby, in each instance analysed, an increase in TLR-9 level is accompanied by the increase in antibody titre. Nevertheless, statistical significance was achieved exclusively for the EBVCA IgA and EBVCA IgG antibodies. More detailed data on this can be found in Supplementary Table S5.

## 3. Discussion

A considerable proportion of the global population is infected with EBV. This infection has been associated with the development of numerous diseases, including cancer. The EBV can enter a latency state, making it a chronic illness. It is the first human tumour virus to be causally associated with a variety of lymphoid and epithelial cancers, including nasopharyngeal carcinoma [24], gastric cancer [24], breast cancer [25], and many other studies have indicated that it is involved in the development of PCa [6,9]. This is based on epidemiological data and EBV gene products that can induce cell transformation and the infection of any cancer cell [26].

The role of EBV in the development of PCa remains uncertain. Nevertheless, convincing evidence points to a pathogenic role for the virus. This is based on epidemiological data and the presence of EBV gene products, which can induce cell transformation in addition to the infection of any tumour cell. The presence of EBV in prostate tissue has been demonstrated in various studies, including those by Nahand [27] and Whitaker [28]. Our previous study showed that EBV was detected in PCa tissue in 49.6% of subjects [23]. In contrast, other studies have failed to detect EBV in PCa tissue, as evidenced by Greisten’s findings [29]. It is crucial to acknowledge that while the presence of EBV DNA may suggest a role for this virus in the development and progression of PCa, it does not provide conclusive evidence to support this hypothesis. It is therefore critical to gain deeper insight into the EBV latent genes’ role in PCa. An awareness of this phenomenon is essential for determining the contribution of viral infections to the development and progression of PCa and for developing more productive strategies to prevent and treat it. 

Despite extensive research, the etiology of PCa is not yet fully understood. However, epidemiological data suggest that age, race, and genetic burden may play a role in the development of PCa [8]. Nevertheless, researchers suggest that EBV infection is one of the causes of PCa development [6,27]. A virus must be demonstrated in tumour cells to be considered an etiological cancer agent. This demonstration must include the presence of the antigen or genome of the virus in the tumour cells. There must be an epidemiological link between the occurrence of cancer and the virus, and the virus isolated from tumour tissue must be capable of transformation in vitro [30,31]. EBV possesses all the above characteristics.

In people with a healthy immune system, EBV can remain asymptomatic for many years. For the process above to occur, EBV-infected cells must be recognized and targeted by the immune system, most probably through the action of antigen-presenting cells (APCs). Then, it stimulates the production of antigen-specific Th lymphocyte responses [26,32]. APCs have several PRR receptors. These include TLRs, nucleotide-binding retinoic acid-inducible receptors, and C-type lectin-like receptors. TLRs play an essential role in the enhanced immune response of the body against pathogens, particularly viruses [33]. Researchers suggest that TLR-9 is required for the innate immune response to DNA virus infection, including EBV [21]. Rapid detection of EBV may be the key to stopping the virus from spreading in the body and controlling the B-lymphocyte infection and latent infection [34].

The precise impact of altered TLR function on cancer risk remains unclear. It is not yet known whether this function should result in an increased or decreased risk of cancer. TLR activation has complex consequences in PCa and the tumour microenvironment. The discovery that TLRs, which are involved in the immune response induced by numerous immune adjuvants, may stimulate anti-tumour immunity has led to the hypothesis that increased TLR activation may have this effect [35]. It can therefore be postulated that a heightened TLR function may impede cancer development.

In contrast, a suppressed TLR activity may enable cancerous cells to avoid detection and elimination by the immune system. Conversely, the activation of TLRs has the potential to stimulate carcinogenesis. This can occur through the following two main mechanisms: firstly, fostering an environment conducive to tumour growth and chemoresistance, and secondly, inducing long-term inflammation and immunosuppression, which facilitates the development and spread of cancerous cells. It can be posited that a decline in activity at the cellular level would result in mitigation against chronic inflammation and an associated reduction in the likelihood of cancerous progression [35]. The activation of TLR-2 and TLR-9 by PCa cells appears to promote tumour growth; conversely, the activation of TLR-3, TLR-5, and TLR-7 has been suggested as a potential way to prevent PCa [36]. In contrast, TLR-4 has been associated with increased and decreased PCa risk [37].

Rex [18] postulates that TLR-2, TLR-3, TLR-7, and TLR-9 are involved in detecting gamma-herpesviruses. In particular, it should be noted that after the transfection of HEK293 cells with TLR-2, UV-inactivated EBV particles strongly induce nuclear factor-kappa B (NF-κB) activation and secretions of monocyte chemoattractant protein (MCP) chemokine-1 [18,38]. Authors have suggested that TLR-2 is currently the only TLR on the cell surface capable of recognizing EBV, although it remains unclear whether this is indeed the case [18]. Additionally, the exact nature of the viral ligands has yet to be convincingly demonstrated, and their role during EBV infection in the human host remains unknown. Furthermore, it is noteworthy that Liu’s [39] investigations into TLR-7 and TLR-9 in various EBV infections have yielded valuable insights. TLR-9 expression was upregulated in monocytes and B lymphocytes in children with chronic active Epstein–Barr virus infection (CAEBV) compared to those with infectious mononucleosis (IM). These results suggest that EBV infection upregulates TLR-9 expression in monocytes. A comparable phenomenon was observed in the case of TLR-7 expression.

Consequently, it is hypothesized that the levels of EBV-associated proteins may influence the expression of TLR-7 and TLR-9. The effects of EBV infection with varying levels of TLR expression may depend on the specificity of the cell. The results obtained by Liu [39] are comparable to those observed in our study, which demonstrated a statistically significant increase in the levels of TLR-2 and TLR-9 in EBV(+) group, with values of 51.36 ng/mL and 13.60 ng/mL, respectively. The levels of TLR-2 and TLR-9 were found to be lower in EBV(−) group (41.07 ng/mL and 11.35 ng/mL) and the Control Group (5.48 ng/mL and 3.81 ng/mL). Conversely, Fathallah’s [40] work demonstrated that EBV inhibits TLR-9 expression primarily through its main oncoprotein, latent membrane proteins 1 (LMP1). This is evidenced by the observation that a mutant EBV that lacks LMP1 has a reduced ability to downregulate TLR-9. Furthermore, they observed reduced TLR-2 transcription and function following an EBV infection of primary B lymphocytes.

Our study also examined the relationship between the TLR levels and the histopathological parameters expressed by the GS. In EBV(+) patients, it was observed that both the TLR-2 and TLR-9 levels showed a tendency to increase with increasing the GS. In contrast, in EBV(−) patients, TLR levels were found to be similar to each other. Statistically significant findings demonstrate the correlation between TLR levels and the presence of EBV. Furthermore, they indicate a statistically significant increase in the TLR levels with the increasing GS score, supporting the conclusion that EBV influences TLR levels. Väisänen et al. [41] reached comparable conclusions by categorizing the material according to the GS, from low-risk to intermediate-risk tumours, compared to high-risk tumours. The study demonstrated significantly higher TLR-9 expression in the most aggressive PCa, those with the highest the GS, compared to tumours with a more favorable prognosis. The results of another study [42] are similar to our findings and those of Väisänen [41], indicating that elevated TLR-9 expression is observed in prostate tumours with higher GS. However, a different conclusion was reached by Mandal et al. [37], who investigated whether there was any possible association between TLR-2, -3, and -9 gene polymorphisms and clinical stages of PCa. Their investigation revealed no significant association between the tumour grade and the TLR-2, -3, and -9 gene polymorphisms. In addition, we investigated whether the T-characteristic had any relationship with TLR levels. The results of our study show that there is a significant increase in the levels of TLRs at the T2 stage, especially in people infected with EBV. Furthermore, the obtained results demonstrate a statistically significant association.

Other autoimmune diseases by gamma-herpesviruses related to TLRs have been investigated in various studies [43,44]. These studies have shown that this effect is mediated by increased levels or signalling of TLRs, indicating a potential role of TLRs in the pathogenesis of these diseases. The findings above are notable for their particularity. They suggest a possible benefit from increased TLR activation regarding infection clearance. We can hypothesize that while TLR activation during gamma-herpesvirus infection of a new host or lytic reactivation has an antiviral effect, activating the pathway may promote disease progression once latency is established. The rationale behind this phenomenon may be attributed to either the expression of inflammatory cytokines (which could lead to an enhanced inflammatory response) or the blocking of reactivation, which would result in an increased likelihood of latently infected cells remaining viable [45]. 

It should also be noted that EBV infection is relatively common. However, only a limited number of individuals develop symptoms due to the infection. It is therefore of the utmost importance to distinguish between the different forms of disease (acute, past, chronic, and reactivation) characterized by different antibody profiles (IgA, IgG, and IgM). It is generally acknowledged that most types of cancer require many years to develop; consequently, the persistence of EBV over such an extended period provides a plausible explanation for the observed contribution of EBV to cancer development in some individuals. Indeed, the EBV genes expressed during the various stages of latent infection have many functions that could contribute to cancer and immune evasion [46]. The reactivation and dissemination of EBV in the host can occur under certain stress conditions. This can result in the progression of the tumour through the facilitation of inflammation and tissue damage [47]. During the lytic/latent stage of infection, infected host cells express different viral antigens, which elicit antigen-specific antibodies. These antibodies reflect the stage of viral infection and the level of host immune response and may therefore serve as biomarkers for EBV-associated malignancies [48]. 

Our previous research evaluated the prevalence of EBV DNA in tissues collected from patients with PCa. Then, we quantified the frequency and levels of EBVCA and EBNA 1 in the IgA and IgG classes [23]. The results obtained in the previous study were utilized in the present investigation, as the study aimed to elucidate the relationship between the TLR-2 and TLR-9 levels and the EBV antibodies (IgA and IgG, respectively, corresponding to EBNA 1 and EBVCA). A correlation was identified between the TLR-2 levels and the anti-EBVCA and anti-EBNA antibodies, with an increase in the antibody titres corresponding to a rise in the TLR-2 levels. Nevertheless, a statistically significant correlation was observed only for the anti-EBVCA IgA and IgG antibodies and the anti-EBNA IgG antibodies. Similar results were achieved for the TLR-2 levels, with the correlation between the TLR-9 levels and the anti-EBVCA and anti-EBNA antibodies. In parallel, a statistically significant correlation was observed between the EBVCA IgA and EBVCA IgG antibodies. 

It should be acknowledged that the study included only 57 EBV(+) patients out of the 115 patients diagnosed with PCa. It is a relatively low number of patients. For this reason, the size of the study cohort is insufficient to allow for any definitive conclusions to be drawn regarding the impact of EBV on PCa. However, an indirect indication of the effect of EBV on PCa may be gleaned from the resulting correlations and antibody levels. Consequently, this study should be regarded as preliminary, and further research is needed to explore this topic in more detail. In particular, including a larger group of patients with EBV(+) PCa in future studies would be beneficial.

## 4. Materials and Methods

### 4.1. Patient Characteristics

The study included 115 men diagnosed with PCa and confirmed according to the European Association of Urology (EAU) classification. Patients were hospitalized at the Department of Urology of the 1st Military Clinical Hospital with an Outpatient Clinic in Lublin between January 2023 and November 2023. The exclusion criteria for the study were patients undergoing chemotherapy or radiotherapy. All patients underwent radical prostatectomy. 

The Control Group consisted of 40 male patients in the hospital clinic. Men suffering from prostate problems and with previous cancer were excluded from the Control Group. The Control Group was required to meet the same age criteria as the study group, which ensured comparability of both groups. All participants completed the survey.

Patients diagnosed with PCa were subjected to statistical analysis according to the selected parameters (Table 1). Additionally, EBV DNA test results for these patients are included. Therefore, patients were divided into the following two groups: the EBV(+) group constituted 49.6% of the cohort, while the EBV(−) group constituted 50.4%. Both groups were not statistically significant in terms of demographic and social characteristics. The analysis also included the Control Group that was similar regarding the analysed characteristics.

### 4.2. Clinical Specimens

The most frequently employed risk stratification methodology incorporates the clinical stage, PSA levels, the GS assessment, and the T-trait (Table 2).

Patients are classified into low-risk group (clinical stage T1–T2a, PSA levels below 10 ng/mL and GS of 6 or less), intermediate-risk group (stage T2b or T1–T2a with PSA levels below 20 ng/mL or GS of 7) and high-risk group (stage ≥T2c or PSA levels of 20 ng/mL or GS of 8 or greater).

### 4.3. Sample Collection

The material consisted of fresh-frozen tumour tissues collected from the patients with PCa. Each sample was assigned a unique identification code thus ensuring patient anonymity. Tissues were collected during surgical procedures and delivered to the laboratory within 24 h. 

Venous blood samples (3–5 mL) were collected according to standard hospital procedure. Blood was collected for routine testing, and the remaining samples were transferred from the hospital laboratory for analysis by our laboratory. The samples were centrifuged at 1500× *g* for 15 min at room temperature, and the serum was separated, ensuring the reliability of our research. 

Tumour tissue and the serum samples were then stored at −80 °C until analysis.

### 4.4. Isolation and Detection of EBV DNA

The isolation and detection of EBV DNA were carried out as previously described [23]. The fresh-frozen tumour tissues were cut and homogenized in a manual homogenizer, Omni TH/Omni International/Kennesewa, GA, USA. DNA was extracted using the QIAamp DNA Mini Kit (Qiagen, Hilden, Germany) as described in the manufacturer’s protocol. To verify the quality of the obtained DNA (presence of inhibitors of the Polymerase Chain Reaction (PCR)), a β-globin assay was performed. The isolated material was subsequently amplified using commercially available GeneProof Epstein–Barr virus PCR Kit (Brno, Czech Republic). All samples and also a negative control were analysed in duplicate. A specific conservative DNA sequence for the EBNA1 was amplified during the PCR process according to the manufacturer’s protocol. The PCR was performed using LightCycler 2.0 Software Version 4.1. (Roche Applied Science System, Penzberg, Germany).

### 4.5. Identification of Antibodies against EBV

The Microblot-Array EBV IgM, IgA, and IgG test kit (TestLine Clinical Diagnostics s.r.o., Brno, Czech Republic) were used to detect anti-EBV antibodies in the IgA, IgM, and IgG classes. It contains a selected combination of specific parts of EBV antigens, namely EBNA1, EBNA2, VCA p18, VCA p23, p54 Early Antigen D (EA-D p54), EA-D p138, EA-R, Rta, ZEBRA, gp85, gp350, and LMP1. The results should be reported in U/mL. Negative results were below 185 U/mL, borderline results were between 185 and 210 U/mL, and positive results were above 210 U/mL. A Microblot-Array reader and software version 2.0.4 were used to read and interpret the results.

### 4.6. TLR-2 and TLR-9

The serum levels of TLR-2 and TLR-9 were quantified using a kit from Cloud-Clone Corp Houston, TX, USA (SEA663Hu and SEA709Mu). The kits were sandwich enzyme immunoassays for the in vitro quantitative measurement of TLR-2 and TLR-9 in human serum, plasma, tissue homogenates, cell lysates, cell culture supernates, and other biological fluids. The concentration of the highly sensitive TLRs tested in the samples was then determined by comparing the O.D. of the samples with a standard curve. The absorbance was measured in a spectrophotometer Labexim Ledetect 96 microplate reader (Lengau, Austria). The results were analysed with the use of a MicroWin 2013 (Lite+) software. The data were presented in terms of ng/mL. The TLR-2 minimum detectable dose of this kit is typically less than 0.112 ng/mL and TLR-9 minimum detectable dose of this kit is typically less than 0.056 ng/mL. 

### 4.7. Statistical Analysis

The results were analysed using GraphPad Prism 10 software version 10.1.0 (San Diego, CA, USA) and Statistica version 13.0. (Krakow, Poland). Categorical variables were expressed as numbers and percentages. The distribution of continuous variables was evaluated using the Shapiro–Wilk test. The baseline characteristics of patients were presented as a percentage. The Pearson’s chi-squared test and Fisher’s exact test were employed to compare the frequency of antibodies in both groups. The Mann–Whitney test or the Kruskal–Wallis test was employed to assess the statistical significance of differences in antibody levels between the two groups. Spearman’s correlation rank test was used to assess the correlation between the TLR-2 and TLR-9 levels and the antibodies. The results were deemed statistically significant at a level of *p* ≤ 0.05.

### 4.8. Ethics

The Lublin Medical University Ethics Committee approved the study and complied with GCP regulations (no. KE-0254/194/10/2022, 6 October 2022). A written informed consent was obtained from each of the participants.

## 5. Conclusions

It should be noted that differences between the results may be due to several factors such as sample size or patient background. It is therefore essential to investigate precisely what the differences may be due to so as to establish the exact correlations. This will help to determine whether TLR polymorphisms can be used as a potential diagnostic or prognostic marker and whether we can develop a new treatment strategy for PCa by targeting TLRs and their signalling pathway. A more detailed examination, encompassing a more significant proportion of the population, is necessary to better understand the relationship between TLRs and PCa. This initial study’s results appear promising, paving the way for further, more in-depth research in this field.

## Figures and Tables

**Table 1:** Analysis of selected parameters of patients with PCa including their division into PCa EBV(+) and PCa EBV(−) and the Control Group.

		EBV				*p*	Total Patients		Control Group		*p*
		Positive (+)		Negative (−)							
		n	%	n	%		n	%	n	%	
Age	54–59	7	6.09	11	9.56	0.3239	18	15.65	6	15.00	0.9218
	60–82	50	43.48	47	40.87		97	84.35	34	85.00	
Place of residence	Urban	37	32.17	31	26.96	0.2112	68	59.13	24	60.00	0.9232
	Rural	20	17.39	27	23.48		47	40.87	16	40.00	
Smoking	Never	12	10.43	10	8.70	0.6034	22	19.13	8	20.00	0.9046
	Ever	45	39.13	48	41.74		93	80.87	32	80.00	
Alcohol abuse	Never	19	16.52	20	17.39	0.4884	39	33.91	14	35.00	0.9653
	≤drink per week	33	28.70	36	31.30		69	60.00	24	60.00	
	>drink per week	5	4.35	2	1.73		7	6.09	2	5.00	
Risk	Low-	20	35.09	34	58.62	0.0026 *					
	Intermediate-	13	22.81	16	27.59						
	High-	24	42.11	8	13.79						
Gleason score	6	20	35.09	34	58.62	0.0052 *					
	7	13	22.81	16	27.59						
	8	11	19.30	2	3.45						
	9	13	22.81	6	10.34						
T	T1	21	36.84	33	56.90	0.0312 *					
	T2	36	63.16	25	43.10						
	T3	0	0.0	0	0.0						
	T4	0	0.0	0	0.0						
N	N0	57	100.0	58	100.0	-					
M	M0	57	100.0	58	100.0	-					

* statistically significant.

**Table 2:** EUA risk groups for PCa [49,50].

Low-Risk	Intermediate-Risk	High-Risk
PSA < 10 ng/mL	PSA 10–20 ng/mL	PSA > 20 ng/mL
GS < 7 (ISUP grade 1)	GS 7 (ISUP grade 2/3)	GS > 7 (ISUP grade 4/5)
cT1-2a	cT2b	cT2c

PSA: prostate-specific antigen and GS: Gleason score.

## Data Availability

Data are contained within the article.

## Supplementary Materials

The following supporting information can be downloaded at: https://www.mdpi.com/article/10.3390/ijms25169053/s1.
