# Supplementary material for: Serum TLR2 and TLR9 in Prostate Cancer Patients in Relation to EBV Status
Source: Int J Mol Sci. 2024 Aug 21;25(16):9053. doi: 10.3390/ijms25169053 (PMC11354572; doi:10.3390/ijms25169053)
Supplement: Supplementary file 1 [file ijms-25-09053-s001.zip › ijms-3091918-supplementary.pdf]

Supplementary Materials Table S1: A comparative analysis of the percentage of TLR-2 and TLR-9 among prostate cancer patients tested for EBV compared to controls.

		EBV(+)						EBV(−)						Control Groups						
**Group**		**n**	**Mean**	**SD**	**Median**	**Min**	**Max**	**n**	**Mean**	**SD**	**Median**	**Min**	**Max**	**n**	**Mean**	**SD**	**Median**	**Min**	**Max**	***p***
TLR-2	Serum	57	51.36	7.81	52.00	37.00	72.30	58	41.07	4.18	42.00	31.20	50.90	40	5.48	1.57	5.25	3.00	8.20	<0.0001*
TLR-9	Serum	57	13.60	1.49	13.80	10.30	17.00	58	1135	145	11.50	8.00	14.00	40	3.81	1.13	3.80	2.00	6.10	<0.0001*

* statistically significant.

Supplementary Materials Table S2: Analysis of TLR-2 and TLR-9 levels among prostate cancer patients in relation to EBV(+) and EBV(−) patients, according to risk groups.

		EBV(+)							EBV(−)						
**Group**		**n**	**Mean**	**SD**	**Median**	**Min**	**Max**	***p***	**n**	**Mean**	**SD**	**Median**	**Min**	**Max**	***p***
TLR-2	Low risk	20	48.09	7.49	47.00	37.00	64.50	0.0003*	34	40.23	3.71	41.50	31.20	41.50	0.1168
	Intermediate risk	13	48.21	4.67	48.00	40.40	58.30		16	42.43	5.37	43.50	31.90	50.90	
	High risk	24	55.79	7.44	55.50	39.10	72.30		8	41.89	2.70	41.50	38.60	45.70	
	p								<0.0001*						
TLR-9	Low risk	20	12.65	1.36	12.35	10.30	16.00	0.0005*	34	11.00	1.56	11.00	8.00	14.00	0.0837
	Intermediate risk	13	13.54	1.08	13.10	12.10	15.80		16	11.95	1.04	12.00	9.00	13.60	
	High risk	24	14.42	1.33	14.20	12.30	17.00		8	11.65	1.32	11.95	10.00	14.00	
	p								<0.0001*						

* statistically significant.

Supplementary Materials Table S3: Analysis of TLR-2 and TLR-9 levels among prostate cancer patients in relation to EBV(+) and EBV(−) patients, according to Gleason score

		EBV(+)							EBV(–)						
**Group**		**n**	**Mean**	**SD**	**Median**	**Min**	**Max**	***p***	**n**	**Mean**	**SD**	**Median**	**Min**	**Max**	***p***
TLR-2	Gleason score 6	20	48.09	7.49	47.00	37.00	64.50	0.0006*	34	40.23	3.71	41.50	31.20	45.70	0.0508
	Gleason score 7	13	48.21	4.67	48.00	40.40	58.30		16	42.43	5.37	43.50	31.90	50.90	
	Gleason score 8	11	52.75	6.49	56.00	39.10	57.20		2	44.40	0.42	44.40	44.10	44.70	
	Gleason score 9	13	58.36	7.44	55.00	50.40	72.30		6	41.05	2.60	40.50	38.60	45.70	
	p							<0.0001*							
TLR-9	Gleason score 6	20	12.65	1.36	12.35	10.30	16.00	0.0013*	34	11.00	1.56	11.00	8.00	14.00	0.1674
	Gleason score 7	13	13.54	1.08	13.10	12.10	15.80		16	11.95	1.04	12.00	9.00	13.60	
	Gleason score 8	11	14.11	1.07	14.20	12.30	15.90		2	11.95	0.07	11.95	11.90	12.00	
	Gleason score 9	13	14.68	1.51	14.50	12.30	17.00		6	11.55	1.54	11.50	10.00	14.00	
	p							<0.0001*							

* statistically significant.

Supplementary Materials Table S4: Analysis of TLR-2 and TLR-9 levels among prostate cancer patients in relation to EBV(+) and EBV(−) patients, according to the T stage

		EBV(+)							EBV(–)						
**Group**		**n**	**Mean**	**SD**	**Median**	**Min**	**Max**	***p***	**n**	**Mean**	**SD**	**Median**	**Min**	**Max**	***p***
TLR-2	T1	21	48.37	7.41	49.00	37.00	64.50	0.0175*	33	40.39	3.65	42.00	31.20	45.70	0.1049
	T2	36	53.10	7.59	54.00	39.10	72.30		25	41.96	4.72	43.00	31.90	43.00	
	p							<0.0001*							
TLR-9	T1	21	12.71	1.35	12.40	10.30	16.00	0.0003*	33	11.03	1.58	11.00	8.00	14.00	0.0571
	T2	36	14.12	1.32	14.10	12.10	17.00		25	11.78	1.13	12.00	9.00	14.00	
	p							<0.0001*							

* statistically significant.

Supplementary Materials Table S5: Spearman's rank correlation analysis in prostate cancer patients

	**R**	**t(N-2)**	**p**
EBVCA p18 IgA & TLR-2	0.5228	3.9749	0.0003*
EBVCA p18 IgG & TLR-2	0.4498	3.3029	0.0019*
EBNA 1 IgA & TLR-2	0.3178	1.9545	0.0589
EBNA 1 IgG & TLR-2	0.4602	3.1099	0.0036*
EBVCA p18 IgA & TLR-9	0.4934	3.6764	0.0007*
EBVCA p18 IgG & TLR-9	0.5478	4.2938	0.0001*
EBNA 1 IgA & TLR-9	0.2471	1.4868	0.1463
EBNA 1 IgG & TLR-9	0.2975	1.8697	0.0697

* statistically significant.
